# Transmission of SARS-CoV-2 in a primary school setting with and without public health measures using real-world contact data: A modelling study

**DOI:** 10.7189/jogh.12.05034

**Published:** 2022-10-01

**Authors:** Lixiang Yan, Stella Talic, Holly Wild, Danijela Gasevic, Dragan Gasević, Dragan Ilic, Joanne Deppeler, Deborah Corrigan, Roberto Martinez-Maldonado, James Trauer

**Affiliations:** 1 Faculty of Information Technology, Monash University, Clayton, Victoria, Australia; 2 School of Public Health and Preventive Medicine, Monash University Clayton, Victoria, Australia; 3 Public Health & Health Sciences, Torrens University Australia, Melbourne, Victoria, Australia; 4 Faculty of Education, Monash University, Clayton, Victoria, Australia; 5 Centre for Global Health, Usher Institute, The University of Edinburgh, Edinburgh, UK; 6 Centre for Learning Analytics at Monash, Monash University, Clayton, Victoria, Australia

## Abstract

**Background:**

Stringent public health measures have been shown to influence the transmission of SARS-CoV-2 within school environments. We investigated the potential transmission of SARS-CoV-2 in a primary school setting with and without public health measures, using fine-grained physical positioning traces captured before the COVID-19 pandemic.

**Methods:**

Approximately 172.63 million position data from 98 students and six teachers from an open-plan primary school were used to predict a potential transmission of SARS-CoV-2 in primary school settings. We first estimated the daily average number of contacts of students and teachers with an infected individual during the incubation period. We then used the Reed-Frost model to estimate the probability of transmission per contact for the SARS-CoV-2 Alpha (B.1.1.7), Delta (B.1.617.2), and Omicron variant (B.1.1.529). Finally, we built a binomial distribution model to estimate the probability of onward transmission in schools with and without public health measures, including face masks and physical distancing.

**Results:**

An infectious student would have 49.1 (95% confidence interval (CI) = 46.1-52.1) contacts with their peers and 2.00 (95% CI = 1.82-2.18) contacts with teachers per day. An infectious teacher would have 47.6 (95% CI = 45.1-50.0) contacts with students and 1.70 (95% CI = 1.48-1.92) contacts with their colleague teachers per day. While the probability of onward SARS-CoV-2 transmission was relatively low for the Alpha and Delta variants, the risk increased for the Omicron variant, especially in the absence of public health measures. Onward teacher-to-student transmission (88.9%, 95% CI = 88.6%-89.1%) and teacher-to-teacher SARS-CoV-2 transmission (98.4%, 95% CI = 98.5%-98.6%) were significantly higher for the Omicron variant without public health measures in place.

**Conclusions:**

Our findings illustrate that, despite a lower frequency of close contacts, teacher-to-teacher close contacts demonstrated a higher risk of transmission per contact of SARS-CoV-2 compared to student-to-student close contacts. This was especially significant with the Omicron variant, with onward transmission more likely occurring from teacher index cases than student index cases. Public health measures (eg, face masks and physical distance) seem essential in reducing the risk of onward transmission within school environments.

The global COVID-19 pandemic, caused by severe acute respiratory syndrome coronavirus 2 (SARS-CoV-2), has rapidly escalated since January 2020 and, as of February 2022, infected over 400 million people and claimed nearly 6 million lives (that we know of) [1]. The world has seen a significant evolution in the dominant SARS-CoV-2 variant of concern over the course of the pandemic, the most recent of which were the Alpha (B.1.1.7), Delta (B.1.6.17.2), and Omicron (B1.1529) variants [2]. These variants have significantly shifted our understanding of the transmission and infection dynamics of SARs-CoV-2, and when compared to previous variants, Delta and Omicron demonstrated increased transmissibility, re-infection rates and infection duration [3-5].

The emergence of the Delta and Omicron variants coincided with high rates of double vaccination in adult populations in many high-income regions, the easing of public health measures (including the re-opening of schools), and the subsequent increase in community transmission of SARS-CoV-2 [4]. Research has consistently demonstrated that children were less likely to contribute to SARS-CoV-2 transmission [6-10], and school environments were deemed generally low risk for the transmission, especially when transmission in broader community was low [8,11,12]. However, the appearance of the Delta and Omicron variants has considerably shifted the narrative on the role of children and adolescents in community transmission of SARS-CoV-2 [13,14], with evidence suggesting higher rates of infection and hospitalisation [15,16], when compared to previous variants [13,17-20]. Increased infection in children has been facilitated by low rates of prior infection immunity due to reduced susceptibility to infection in previous COVID-19 waves [18] and low rates of double and triple vaccination in this age group [17,19,21]. The Delta and Omicron variants demonstrated considerably higher transmissibility and immune evasion, from both previous infection and vaccination [22-24], reinforcing the continued importance of effective public health measures, such as face masks [13,25,26] and physical distancing [27,28] as key risk prevention strategies alongside vaccination to reduce community transmission [2,4,5,21,29-32].

Understanding the probability of transmission of different variants, as well as the potential efficacy of public health measures in school settings is essential to predicting and mitigating the risk of transmission for staff and students. To understand this probability, we conducted an exploratory observational study to model the likelihood of SARS-CoV-2 transmission using indoor positioning tracking data collected prior to the pandemic. By using exact students’ and teachers’ physical positioning traces, we aimed to estimate the frequency and distribution of close contacts that occurred daily between students and teachers, the probability of transmission of the Delta and Omicron variants compared to the original Alpha variant, as well as the likelihood of SARS-CoV-2 transmission within an open-plan primary school environment. This is particularly relevant for open-plan learning settings where several classes share the same physical space, and children can freely interact with peers and teachers from their classes and other classes [33].

## METHODS

### Participants

A total of 104 participants, including 98 6th year students (49 males, 47 females, and 2 unidentified) and six teachers (two female and two male full-time teachers, one part-time male teacher, and one female aide) from an Australian open-plan, primary school participated in the current study through opportunity sampling. Informed consent was obtained from both teachers and the parents of students. Ethics approval was received from the Department of Education and Training of the State of Victoria in Australia (Project ID: 2018_003877) and Monash University (Project ID: 16615).

### Materials

**Figure 1:**
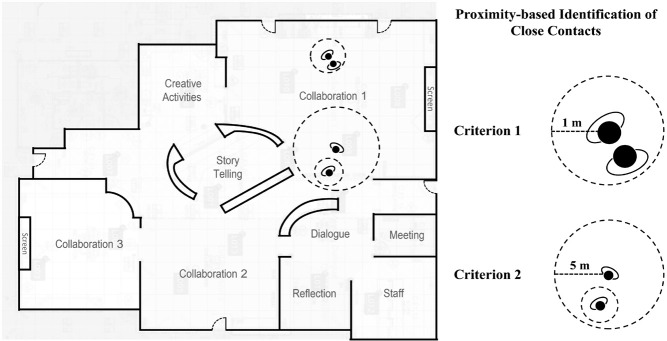
The learning spaces and examples of the proximity-based identification method of close contacts under each criterion. Criterion 1 represents contacts within one-meter proximity for more than 15 minutes. Criterion 2 represents contacts within 5 m proximity for more than 40 minutes.

Each participant was assigned a Bluetooth Low Energy (BLE) Quuppa positioning tag that uses Bluetooth 5.1 Standard technology; each student wore a Tatwah Mango BLEWB200 wristband, and each teacher wore a Jeewey JW-C1809C card tag. Fourteen Quuppa LD6-L locators were installed in the building, a large open area segmented into different learning spaces (**Figure 1**) through which children could freely move. The position of the participants was sampled every 200 milliseconds, and the location was calculated through the Angle-of-Arrive method. The latency of this system is around 200 milliseconds, and the accuracy is approximately 20 cm. An illustration of the positioning tracking software is available in the **Online Supplement Document** (Figure S1).

### Data collection

Data were collected over eight weeks, from July 22 to September 13, 2019. From Monday to Friday, the teachers distributed the positioning tags to students when they arrived at school and collected them back at the end of the day. Students and teachers wore the tag for six hours and twenty minutes each day, from 9:00 am to 3:20 pm In instances when students and teachers were outside the tracking area, outside the classroom (eg, toilet, recess, and lunch break), or recording was temporarily interrupted (signal interference), data were not registered.

### Analysis

We used the Spyder IDE [34] and the NumPy [35] software package for the analysis. A random week (August 26 to August 30, 2019) was chosen from the recorded data for the current analysis using Python’s random choice function, as this is approximately equivalent to the incubation period of COVID-19 (the period between a primary case-patient (infector’s) symptom onset and a secondary case-patient (infectee’s) symptom onset) [36]. Three separate analyses were performed. First, we extracted the number of close contacts among participants from their physical positioning traces and conducted social network analysis to explore the similarities and differences between the distribution of contacts concerning two different definitions (elaborated in the following subsection). Second, we estimated the probability of transmission per contact for different variants of SARS-CoV-2 under different public health measures. Finally, we modelled the probability of onward transmission occurring in primary school using the secondary attack rates from Macartney et al.’s study [8] for the Alpha variant, the reports of the National Centre for Immunisation Research and Surveillance (NCIRS) [37] for the Delta variant, and WHO’s most recent report [3] and the preliminary findings of the UK Health Security Agency [38] for the Omicron variant. Each of these analyses is elaborated in detail in the following subsections.

#### Close contacts

Close contacts were identified using a proximity-based identification method relying on the Euclidean distances between two participants [39,40]. Two different criteria items were used, both adapted from the guidelines of the Department of Health and Human Services in Victoria [41], which have also been applied in a recent COVID-19 study with Australian children [8]. A meaningful contact occurred when two participants met either criterion 1 (being within one-meter proximity of one another for more than 15 minutes) or criterion 2 (being within 5 m proximity of one another for more than 40 minutes) (**Figure 1**). Four different types of contacts were modelled from the data (**Table 1**). Student-to-student contacts (S-S) and student-to-teacher contacts (S-T) were defined as contacts between a student index case and other susceptible students or teachers, respectively. In contrast, teacher-to-student (T-S) and teacher-to-teacher (T-T) contacts were defined as contacts between a teacher index case and other susceptible students or teachers, respectively. The daily mean and the 95% confidence interval (CI) were calculated for each type of contact using the aforementioned close contacts criteria. We also aggregated the number of contacts identified through close contact criteria because satisfying either criterion would expose students or teachers to potential infection [8].

**Table 1:** Secondary attack rates (r) and 95% CI for the three SARS-CoV-2 variants

SARS-CoV-2 Variants	Primary Case	Close Contact	r (%)	Lower CI (%)	Upper CI (%)
Alpha	Student	Student	0.31	0.04	1.11
	Student	Teacher	0.97	0.02	5.29
	Teacher	Student	1.49	0.65	2.92
	Teacher	Teacher	4.38	1.78	8.81
Delta	Student	Student	1.63	0.95	2.60
	Student	Teacher	1.46	0.40	3.70
	Teacher	Student	6.96	5.22	9.05
	Teacher	Teacher	11.2	7.85	15.4
Omicron	Student	Student	3.45	2.43	4.75
	Student	Teacher	2.92	1.27	5.67
	Teacher	Student	14.5	12.0	17.2
	Teacher	Teacher	23.5	18.7	28.7

CI – confidence interval, r – secondary attack rates

Social network analysis was performed to illustrate the distribution of close contacts concerning criteria 1 and 2 within the open-plan learning space. Five social network graphs were produced for each criterion, representing close contacts from Monday to Friday. Each node in the resulting social networks represented a student or a teacher. A line connecting two nodes represented close contact detected among these teachers/students. The Fruchterman-Reingold force-directed algorithm (spring layout) was used to adjust the network layout by clustering well-connected nodes together. Degree distribution graphs were used to illustrate the number of individuals students and teachers had contact with during a day.

#### Probability of transmission per contact

We derived the probability of transmission per contact (*p*) by applying the Reed-Frost model that ensures that the per-contact risk and the total risk of infection can be expressed as probabilities. The following formula was based on three input values: *n* – average daily contacts with an infected individual during the incubation period (mean of the aggregated contacts from the prior analysis), *r* – secondary attack rate, and *m* – a multiplier that represents the escalated risk of infection when specific mitigation measures were removed:



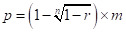



We considered three sets of secondary attack rates, corresponding to the original SARS-CoV-2 Alpha variant (B.1.1.7), the Delta variant (B.1.617.2), and the Omicron variant (B.1.1.529) based on the WHO designated variants of concern list [42]. **Table 1** presents these secondary attack rates and their 95% CI. The secondary attack rates for the Alpha and Delta variant were obtained from studies conducted in Australian primary schools [8,37]. Namely, the first set (Alpha) was defined based on the results of Macartney et al.’s study [8], which was conducted in the early onset of the pandemic, from January 25 to May 1, 2020. The second set (Delta) was defined based on the reports of the National Centre for Immunisation Research and Surveillance (NCIRS) [37], which reported the local transmission of the Delta variant from June 16 to August 19, 2021. The third set (Omicron) was defined based on WHO’s most recent report [3] and the preliminary findings of the UK Health Security Agency [38], given that only a limited number of reliable peer-reviewed studies have been published regarding the transmission rate of the Omicron variant in school settings. The risk of close contact of an Omicron index case becoming a secondary case was 2.09 (95% CI = 1.54-2.79) times higher than that of the Delta variant [3,38] and 11.1 times higher than that of the Alpha variant.

The multiplier (*m*) was introduced to estimate the risk of infection if public health measures were relaxed in schools. The aforementioned secondary attack rates were identified or estimated based on studies conducted after introducing state-wide public health measures in New South Wales (NSW), Australia [8,37]. These measures involved wearing face masks and physical distancing [37]. Leung et al. [43] estimated that the efficacy of surgical face masks in reducing coronavirus RNA in aerosols and respiratory droplets was approximately 40%. Another study [44] also observed that the risk of infection was around 2.83 times higher if mitigation measures (particularly face masks and physical distancing) were absent. To the best of our knowledge, there is little empirical evidence on the efficacy of physical distancing, specifically restricting a minimum social distance of 1.5 m [37], in reducing the risk of infection, alone. Therefore, based on the estimations and findings of these studies [43,44], we used three different values for the m parameter in our model (**Table 2**).

**Table 2:** Risk of infection under different public health measures

*m*	Public health measures
1.00	Face mask and physical distancing
1.67	Physical distancing but no face mask
2.83	No public health measures

*m* – multiplier

#### Modelling SARS-CoV-2 transmission in a primary school setting

The binomial distribution was used to model the probability of onward transmission occurring if 1) one infectious student or 2) one infectious teacher was in the learning space at the beginning of the week. Onward transmission was defined as one or more additional infections occurring over a school week with a single index case at the beginning of the week [45]. A total of 12 simulations were conducted, each representing a combination of the transmission type (4 types; **Table 1**) and the public health measures (3 settings; **Table 2**). For each simulation, we modelled the probability of onward transmission for each of the three variants of SARS-CoV-2 (**Table 1**) based on the probabilities of transmission per contact (*p*) parameter and the average daily contacts with the infected individual (*n*) parameter, both of which were calculated in the previous steps. The results were summarised into 12-line graphs where the *x-axis* represents the number of days after the index case was introduced to the school, and the *y-axis* represents the probability of onward transmission with and without public health measures. The Python code for this modelling analysis is available in the Open Science Framework (https://osf.io/rgeb2/?view_only=ffe77156bc72449cbf3f7403ea5c7835).

## RESULTS

### Number of close contacts

**Figure 2:**
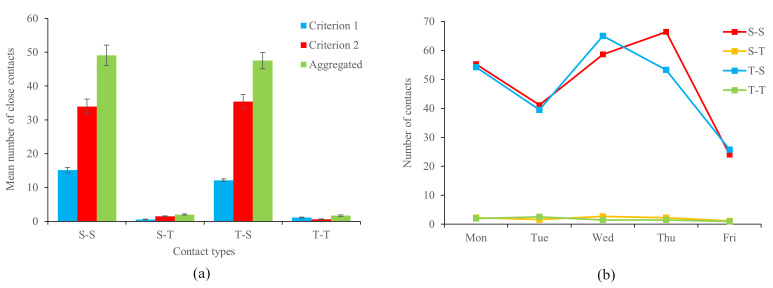
**Panel A.** The mean and 95% confidence interval for the number of close contacts during the period between August 26 to August 30, 2019. **Panel B.** Change in the number of contacts during a week. Criterion 1 represents contacts within one-meter proximity for more than 15 minutes. Criterion 2 represents contacts within 5 m proximity for more than 40 minutes. Student-to-student contacts (S-S) and student-to-teacher contacts (S-T) were contacts between a student index case and other susceptible students or teachers, respectively. Teacher-to-student (T-S) and teacher-to-teacher (T-T) contacts were contacts between a teacher index case and other susceptible students or teachers, respectively.

On average, student index cases had around 15.1 close contacts (95% CI = 14.3-16.0) with other students and 0.54 close contacts (95% CI = 0.47-0.62) with teachers meeting criterion 1 (within one-meter proximity for over 15 minutes; **Figure 2**, Panel A, blue bars). For the same criterion, a teacher index case would have around 12.2 close contacts (95% CI = 11.8-12.5) with students and 1.10 close contacts (95% CI = 0.99-1.21) with other teachers.

Considering criterion 2 (within 5 m proximity for more than 40 minutes, **Figure 2**, Panel A, red bars), there were 34.0 S-S close contacts (95% CI = 31.8-36.2), 1.46 S-T close contacts (95% CI = 1.35-1.57), 35.4 T-S close contacts (95% CI = 33.4-37.5), and 0.60 T-T close contacts (95% CI = 0.48-0.72).

The aggregate mean number of close contacts was calculated as contacts meeting either of the two criteria, within one-meter proximity for more than 15 minutes or within 5 m proximity for more than 40 minutes. The aggregated mean numbers of S-S and S-T close contacts were 49.1 (95% CI = 46.1-52.1) and 2.00 (95% CI = 1.82-2.18), respectively, while the mean numbers of T-S and T-T close contacts were 47.6 (95% CI = 45.1-50.0) and 1.70 (95% CI = 1.48-1.92), respectively (**Figure 2** **Panel A**, green bars). Additionally, aggregated close contacts with students (mean contact’s standard deviation was 16.7 for S-S and 15.2 for T-S) exhibited a great extent of temporal variation. In contrast, close contacts with teachers remained stable at a lower level (mean contact’s standard deviation was 0.62 for S-T and 0.57 for T-T) (**Figure 2**, Panel B).

**Figure 3:**
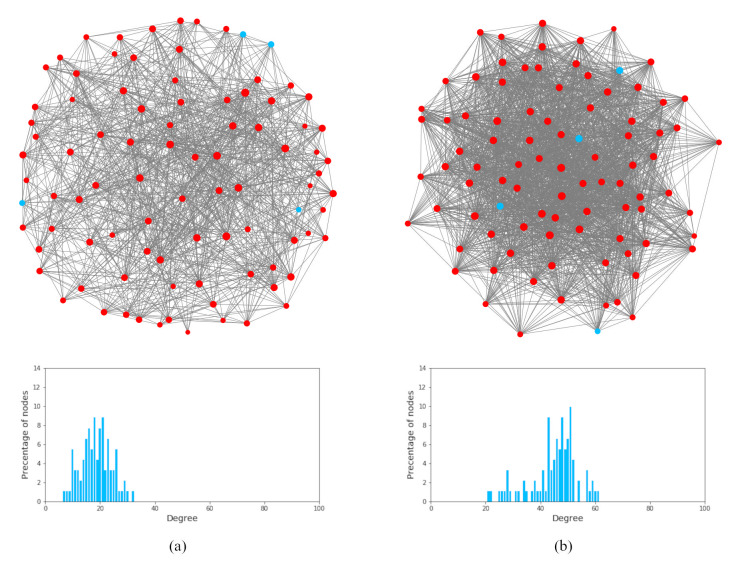
**Panel A.** Social networks and degree distribution of close contacts concerning Criterion 1 (within one-meter proximity for more than 15 minutes) on August 26, 2019. **Panel B.** Social networks and degree distribution of close contacts concerning Criterion 2 (within 5 m proximity for more than 40 minutes) on August 26, 2019. Blue nodes represent teachers and red nodes represent students.

**Figure 3** illustrates the social networks and degree distributions of close contacts in the learning spaces on August 26, 2019. The social networks revealed that the distribution of contacts meeting criterion 2 (**Figure 3**, Panel B) was denser than that of the social networks meeting criterion 1 (**Figure 3**, Panel A). The mean density of contacts in the social network derived from criterion 2 was 0.47, which suggests that each individual in the learning space had close contact with, on average, 47% of the cohort. This proportion was more than twice greater than the density of contacts in the social network derived from criterion 1 (0.20). The layout of both social networks suggested that close contacts in the learning space were occurring in one large cluster instead of being segmented into small components. The degree distributions of the social network derived from Criterion 1 were similar to a normal distribution, whereas the degree distributions of the social network derived from Criterion 2 were more negatively skewed.

### Probability of transmission per close contact

**Figure 4:**
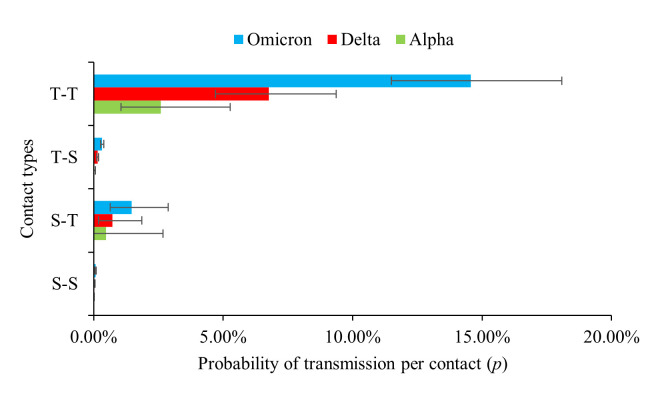
The probability and confidence interval of transmission per contact for S-S (contacts between a student index case and other susceptible students), S-T (contacts between a student index case and other susceptible teachers), T-S (contacts between a teacher index case and other susceptible students), and T-T (contacts between a teacher index case and other susceptible teachers) contacts for the three variants of SARS-CoV-2.

As shown in **Figure 4**, for all three variants of SARS-CoV-2, risk of transmission per contact in students remained generally low. For the Alpha variant, the probability of transmission per close contact (p) for S-S contacts was 0.00633% (95% CI = 0.000815%-0.0227%), and for T-S contacts was 0.0316% (95% CI = 0.0137%-0.0623%). For the Delta variant, the p for S-S contacts was 0.0335% (95% CI = 0.0194%-0.0537%) and 0.152% (95% CI = 0.113%-0.199%) for T-S contacts. For the Omicron variant, the p for S-S contacts was 0.0715% (95% CI = 0.0501%-0.0991%) and 0.328% (95% CI = 0.268%-0.397%) for T-S contacts. By contrast, per-contact transmission risk to teachers was more likely to occur in both student and teacher index cases. For the Alpha, Delta, and Omicron variants, the probability for S-T contacts were 0.486% (95% CI = 0.0100%-2.68%), 0.733% (95% CI = 0.200%-1.87%), and 1.47% (95% CI = 0.637%-2.88%), respectively, while for T-T contacts, it was 2.60% (95% CI = 1.05%-5.28%), 6.76% (95% CI = 4.70%-9.37%), and 14.6% (95% CI = 11.5%-18.1%), respectively.

### Modelling the transmission of SARS-CoV-2 in a primary school setting with and without public health measures

**Figure 5:**
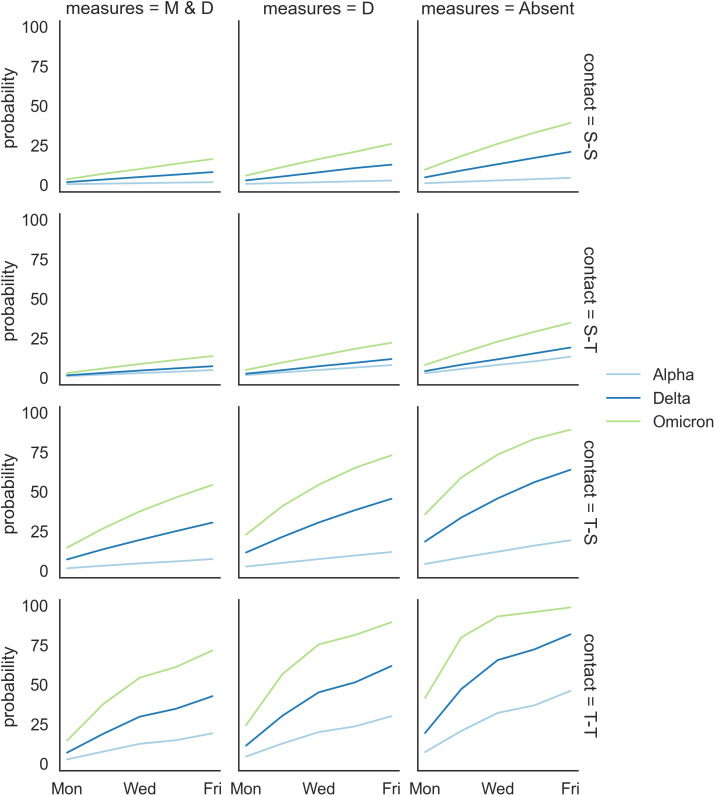
The probability of onward transmission occurring in schools under different public health measures and different SARS-CoV-2 variants, where M represents mandatory mask requirements, D represents physical distancing, and Absent represents neither of these two public health measures in place.

The results of the modelling of the SARS-CoV-2 transmission with and without public health measures are outlined in **Figure 5** and Table S1 in the **Online Supplementary Document**. The probability of onward S-S and S-T SARS-CoV-2 transmission was relatively low across the SARS-CoV-2 variants if a student index case was introduced to schools with state-wide public health measures (mandatory mask and physical distancing) [8,37]. In case of mandatory mask requirements being removed from the school setting with physical distancing in place, the probability of onward S-S and S-T SARS-CoV-2 transmission would increase but remain at a relative low range (under 30%) across the SARS-CoV-2 variants. Although these probabilities could further increase to around 40% (for the Omicron variant) if mandatory mask and physical distancing requirements are not in place in school settings, they were still considerably lower than the probability of onward virus transmission with a teacher index case, results being consistent across the SARS-CoV-2 variants. In particular, the probability of onward T-S and T-T transmission in schools with state-wide public health measures (mandatory mask and physical distancing) [8,37] already surpassed the highest probability of onward virus transmission with a student index case (when both mandatory mask and physical distancing requirements are not in place in school settings) across the SARS-CoV-2 variants. The absence of the mandatory mask requirements, with physical distancing in place, could further increase the probability of onward T-S transmission and T-T transmission to a concerning level (mostly over 50%), especially for the Delta and Omicron variant. The probability of onward T-S transmission and T-T transmission in schools with the absence of both mandatory mask and physical distancing requirements was almost inevitable for the Omicron variant.

## DISCUSSION

Using students’ and teachers’ physical positioning traces, we illustrated that close contacts in open-plan learning spaces occurred within a single large cluster. The density of these contacts depended on the definition of “meaningful contact”. In particular, the distance aspect of the criteria used in this study appeared to be more important than the duration of contact since more contacts were identified as the distance aspect of the criteria loosened from one (criterion 1) to five meters (criterion 2), while the duration criteria tightened from 15 minutes (criterion 1) to 40 minutes (criterion 2). We estimated that the average daily close contact with an infected individual was most frequent in the forms of S-S- and T-S contact, whereas S-T and T-T close contacts were relatively rare. Despite the low frequency of T-T contacts, we estimate that teachers were still subject to a higher risk of per-contact transmission than students, especially from a teacher index case and with the Omicron variant; while the per-contact risk of transmission was considerably lower for students, even with the Omicron variant. The findings of the transmission modelling suggested that onward transmission was more likely to occur with a teacher index case than a student index case. Onward T-S transmission and T-T transmission were almost inevitable for the Omicron variant in schools without public health measures in place. Therefore, public health measures appear to be essential in reducing the risk of onward transmission in schools

The results of our study indicate that in pre-pandemic schools the probability of SARS-CoV-2 infection is highly dependent on the variant. The Delta and Omicron SARS-CoV-2 variants demonstrated significantly higher infection growth rates than previous variants, resulting in the increased community transmission of SARS-CoV-2 globally [46,47]. Transmission data in household [46] and hotel quarantine studies [48,49] on the Omicron variant suggest that it is significantly more infectious than the Delta variant [49-51], and both variants demonstrated high rates of immune evasion, lowered vaccine efficacy, and increased re-infection within the community [3,46,52]. The accelerated community transmission of these variants has been facilitated by the easing of the community-based public health measures in many regions [3,46,52]. Importantly, studies on previous SARS-CoV-2 variants have suggested that community-based mitigation factors have proven to important in regard to school-based COVID-19 outbreaks; with school-based cohort studies in Australia [8] and Singapore [53] conducted in areas of low transmission, observing very low likelihood of both primary and secondary SARS-CoV-2 transmission. Conversely, in areas with high community transmission, school-based outbreaks have been demonstrated more frequently [6]. Current high rates of community transmission of SARS-CoV-2 are likely to increase the risk of SARS-CoV-2 transmission within school environments, and while there is currently no observational research on the risk of transmission of Omicron within schools, increased school transmission of the Delta variant has been reported in the UK [47] and Australia [37], as such, risk reduction measures within schools will be key to ensuring a safe working environment for students and teachers. Our results extend available evidence by providing information on the efficacy of both individual and combined public health measures within school environments to reduce SARS-CoV-2 transmission.

Our study also indicates a significantly increased risk of student-to-student (S-S), student-to-teacher (S-T), and teacher-to-student (T-S) transmission associated with both the Delta and Omicron variants comparatively to the Alpha variant. These results have been mirrored in growing daily case numbers in young people, with a marked increase in daily infections in children between June 2021 and February 2022 in the UK [54], increasing from 0.2% to 11.5% in Year 2-6 students, and from 0.5% to 8.5% for those in Years 7-11 [16]. This increased risk of infection in students is likely facilitated by increased community transmission, lower rates of double and triple vaccination in children when compared to adults [17,19,21,55], and low rates of prior infection immunity [56]. Current evidence suggests a slightly higher symptom burden in children associated with Delta infection [57] [56] though rates of Delta and Omicron hospitalisation in children remain similar to previous variants [57,58]. While research is still ongoing, preliminary findings on prolonged illness duration (ie, long COVID) in children, demonstrate that the risk remains low with the Delta infection [59].

Delta, however, does present an increased risk of severe disease and hospitalisation for both adults and children with co-morbidities when compared to Omicron and previous variants [18,56,57,60]. Our results demonstrate a significantly increased risk of teacher-to-student (T-S), and teacher-to-teacher (T-T) transmission. Research on previous SARS-CoV-2 variants within school settings in both Australia [8] and the UK [11] have noted that teachers are more at risk of contracting COVID-19 disease in the school settings than students. Macartney et al. [8] demonstrated that the rate of transmission (4.4%) was more frequent than those of student to staff (1.0%), staff to student (1.5%) and student to student (0.3%). Similarly, Ismail et al. [11] also noted a higher number of index cases in teachers/staff in comparison to students, and of the 58 primary cases in teachers/staff, two were hospitalised and required intensive care, and one died. The results of our study are consistent with the above-mentioned ones, and we build on the available evidence by reporting higher transmission from teacher index cases of all variants, which is particularly true for the Omicron variant that demonstrated a significantly higher probability of transmission from T-S and T-T compared to other variants. Importantly, this risk, while not eliminated, was substantially reduced in the presence of public health measures.

In many of the school-based cohort studies on previous variants, especially those that observed low to no virus transmission [8,53], a combination of stringent public health measures, such as physical distancing and smaller class sizes were used within educational facilities. This study emphasizes the importance of public health measures within school environments as a key to the safe reopening of schools [61] and other educational environments. However, public health measures within educational facilities may come with significant costs and, if required in the longer term, may place a significant strain on school budgets. US research has estimated that an additional cost of between $55 and $442 per student per year is required to implement public health measures in schools [62]. Alternatively, targeted public health measures for teachers/staff could potentially reduce such costs while ensuring the safety of the more vulnerable population in school environments [61]. The indoor positioning data we have used in our modelling represent the physical dynamics of students’ and teachers’ interactions prior to the COVID-19 pandemic. These data provide potential insights into both the efficacy of school-based public health measures, as well as outline who can be most at risk within educational environments in the absence of public health measures.

One of our study limitaitons is that the tracking system used to identify close contacts only captured students’ and teachers’ physical positioning traces within the building area (**Figure 1**). Close contacts during recess and lunchtime at external facilities (eg, bathrooms and the playground) were not registered. This missing information may have led to underestimation of the actual number of close contacts. Furthermore, the data we analysed are generalisable to open-plan learning spaces with a large student population and may be less relevant to traditional lecture-style classrooms, where less opportunity for close contact is expected and less student mobility. Additionally, while positioning data can be used to identify close contacts that occurred within the learning spaces, the nature of the contacts remains unknown. For example, we did not consider participants’ body orientation during close contact. In instances where participants were sitting back-to-back with each other, the risk of transmission from droplets and airborne exposure could be lower than in face-to-face scenarios. Many school environments have incorporated ventilation-based measures to reduce the risk of SARS-COV-2 transmission. There are currently no data on the efficacy of this measure within schools or similar high-risk environments, so we were unable to include the effects of these measures within our model.

Despite the limitations, using physical positioning traces to model disease transmission has unique advantages over traditional data collection methods, such as interviews, surveys, and direct observations. These trace data are less susceptible to recall bias and provide higher spatial and temporal resolution, which can enable the analysis of both contact duration and frequency [40]. The non-intrusive and automated nature of our positioning tracking approach also ensured the authenticity of students’ and teachers’ close contact data, reducing the possible influence of observational effects. Consequently, results generated from these authentic data could be more representative than computer simulation studies and can motivate the punctual use of similar sensing technologies to model human behaviours in authentic physical spaces, with integrity [39].

## CONCLUSIONS

We investigated the potential transmission of SARS-CoV-2 in a primary school setting with and without the use of public health measures, using fine-grained physical positioning traces captured before the COVID-19 pandemic. Our findings illustrated that despite a lower frequency of close contacts, teacher-to-teacher close contacts demonstrated a significantly higher risk of per contact transmission of SARS-CoV-2 compared to student-to-student close contacts. This was especially significant with the Omicron variant. Onward transmission was also more likely to occur from teacher index cases than student index cases with public health measures being essential in reducing the risk of onward transmission within school environments.

## Additional material


Online Supplementary Document
