# Supplementary material for: Transmission of SARS-CoV-2 in a primary school setting with and without public health measures using real-world contact data: A modelling study
Source: J Glob Health. 2022 Oct 1;12:05034. doi: 10.7189/jogh.12.05034 (PMC9526455; doi:10.7189/jogh.12.05034)
Supplement: Online Supplementary Document [file jogh-12-05034-s001.pdf]

Online Supplementary Document

**Table S1:** The probability of onward transmission occurring in schools under different public health measures and different SARS-CoV-2 variants.	Page 2
**Figure S1:** The learning spaces and illustration of the tracking system where the green and purple dots represent students and teachers, respectively. The green and purple lines represent students and teachers' movement trajectories, respectively.	Page 3

Table S1: The probability of onward transmission occurring in schools under different public health measures and different SARS-CoV-2 variants.

SARS-CoV-2 Variants	Contact	Measures	Probability (%)	Lower CI (%)	Upper CI (%)
**Alpha**	S-S	M & D	1.53	1.46	1.60
		D	2.59	2.49	2.69
		Absent	4.30	4.16	4.44
	S-T	M & D	4.76	4.65	4.87
		D	7.92	7.72	8.11
		Absent	13.2	13.0	13.4
	T-S	M & D	7.28	7.09	7.47
		D	11.7	11.5	11.9
		Absent	19.1	18.9	19.3
	T-T	M & D	19.0	18.8	19.3
		D	29.9	29.6	30.2
		Absent	45.8	45.5	46.2
**Delta**	S-S	M & D	7.93	7.77	8.10
		D	12.6	12.4	12.8
		Absent	20.7	20.5	20.9
	S-T	M & D	7.17	7.00	7.34
		D	11.7	11.5	11.9
		Absent	19.0	18.7	19.2
	T-S	M & D	30.3	30.0	30.6
		D	45.4	45.1	45.7
		Absent	63.7	63.4	64.0
	T-T	M & D	42.5	42.2	42.9
		D	61.6	61.3	61.9
		Absent	81.6	81.4	81.9
**Omicron**	S-S	M & D	16.2	16.0	16.4
		D	25.8	25.5	26.0
		Absent	39.0	38.7	39.3
	S-T	M & D	13.6	13.4	13.8
		D	22.0	21.7	22.2
		Absent	34.6	34.4	34.9
	T-S	M & D	54.1	53.8	54.4
		D	72.8	72.5	73.1
		Absent	89.0	88.8	89.2
	T-T	M & D	71.4	71.2	71.7
		D	89.2	89.0	89.4
		Absent	98.6	98.5	98.7

M – mandatory mask, D – physical distancing, Absent – neither of mandatory mask nor physical distancing in place, S-S – contacts between a student index case and other susceptible students, S-T – contacts between a student index case and other susceptible

teachers, T-S – contacts between a teacher index case and other susceptible students, and T-T – contacts between a teacher index case and other susceptible teachers.

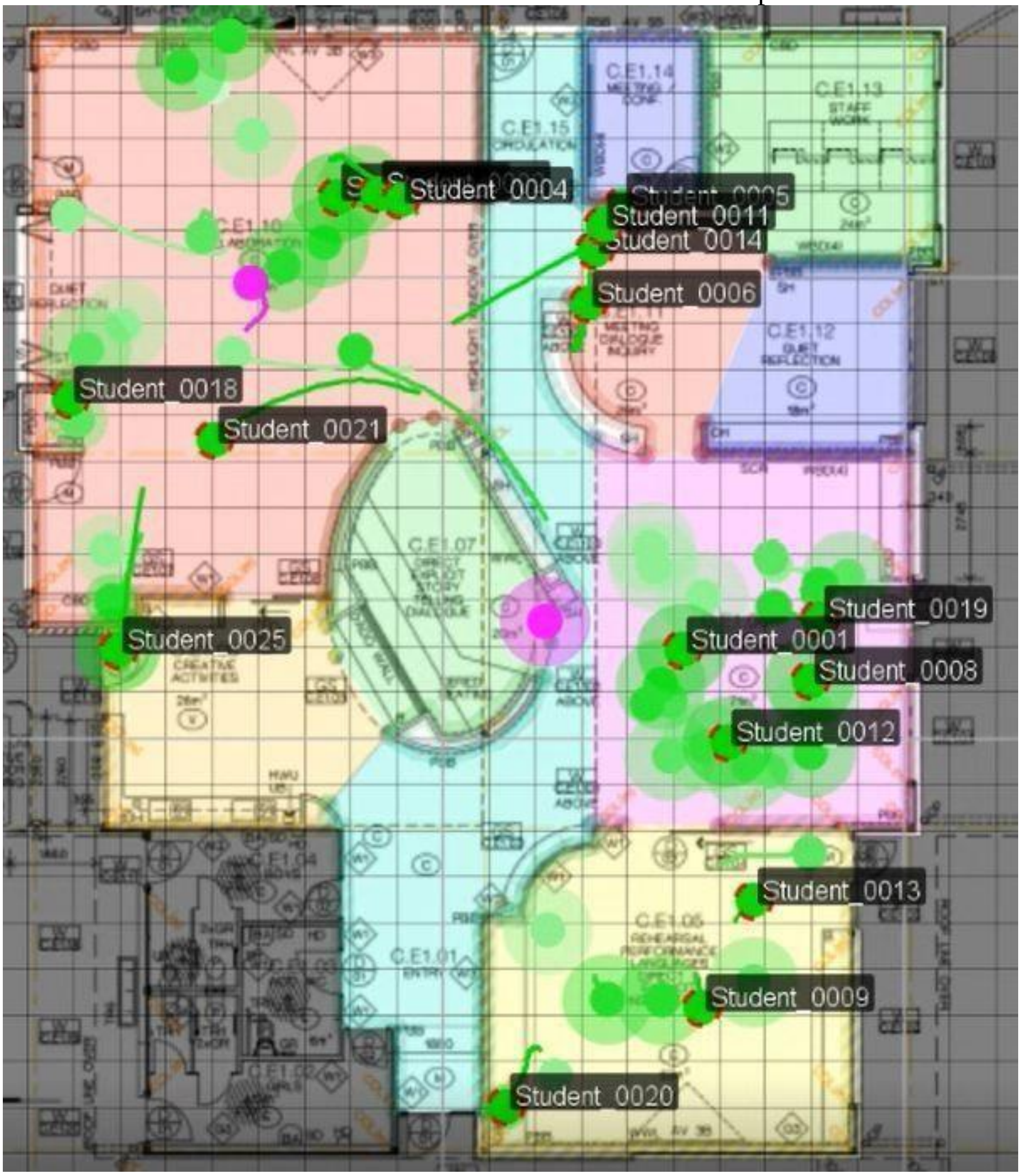

Figure S1: The learning spaces and illustration of the tracking system where the green and purple dots represent students and teachers, respectively. The green and purple lines represent students and teachers' movement trajectories, respectively.
